# Genetic Diversity and Phylogenetic Relationships of Coevolving Symbiont-Harboring Insect Trypanosomatids, and Their Neotropical Dispersal by Invader African Blowflies (Calliphoridae)

**DOI:** 10.3389/fmicb.2018.00131

**Published:** 2018-02-07

**Authors:** Tarcilla C. Borghesan, Marta Campaner, Tania E. Matsumoto, Omar A. Espinosa, Victor Razafindranaivo, Fernando Paiva, Julio C. Carranza, Nestor Añez, Luis Neves, Marta M. G. Teixeira, Erney P. Camargo

**Affiliations:** ^1^Department of Parasitology, Institute of Biomedical Sciences, University of São Paulo, São Paulo, Brazil; ^2^Department of Entomology, University of Antananarivo, Antananarivo, Madagascar; ^3^Centro de Ciências Biológicas e da Saúde, Universidade Federal do Mato Grosso do Sul, Campo Grande, Brazil; ^4^Laboratorio de Investigaciones en Parasitología Tropical (LIPT), University of Tolima, Ibagué, Colombia; ^5^Department of Parasitology, University of Los Andes, Mérida, Venezuela; ^6^Centro de Biotecnologia, Eduardo Mondlane University, Maputo, Mozambique; ^7^Department of Veterinary Tropical Diseases, Faculty of Veterinary Science, University of Pretoria, Pretoria, South Africa

**Keywords:** endosymbiont, reduced genomes, fly parasites, transoceanic dispersal, co-evolution, phylogeography, parasite spillover, DNA barcoding

## Abstract

This study is about the inter- and intra-specific genetic diversity of trypanosomatids of the genus *Angomonas*, and their association with Calliphoridae (blowflies) in Neotropical and Afrotropical regions. Microscopic examination of 3,900 flies of various families, mostly Calliphoridae, revealed that 31% of them harbored trypanosomatids. Small subunit rRNA (SSU rRNA) barcoding showed that *Angomonas* predominated (46%) over the other common trypanosomatids of blowflies of genera *Herpetomonas* and *Wallacemonas*. Among *Angomonas* spp., *A. deanei* was much more common than the two-other species, *A. desouzai* and *A. ambiguus*. Phylogenetic analyses based on SSU rRNA, glycosomal glyceraldehyde-3-phosphate dehydrogenase (gGAPDH) and internal transcribed spacer rDNA (ITS rDNA) sequences revealed a marked genetic diversity within *A. deanei*, which comprised four infraspecific genotypes (Dea1–Dea4), and four corresponding symbiont genotypes (Kcr1–Kcr4). Host and symbiont phylogenies were highly congruent corroborating their co-divergence, consistent with host-symbiont interdependent metabolism and symbiont reduced genomes shaped by a long coevolutionary history. We compared the diversity of *Angomonas/*symbionts from three genera of blowflies, *Lucilia, Chrysomya* and *Cochliomyia. A. deanei, A. desouzai*, and *A. ambiguus* were found in the three genera of blowflies in South America. In Africa, *A. deanei* and *A. ambiguus* were identified in *Chrysomya*. The absence of *A. desouzai* in Africa and its presence in Neotropical *Cochliomyia* and *Lucilia* suggests parasite spillback of *A. desouzai* into C*hrysomya*, which was most likely introduced four decades ago from Africa into the Neotropic. The absence of correlation between parasite diversity and geographic and genetic distances, with identical genotypes of *A. deanei* found in the Neotropic and Afrotropic, is consistent with disjunct distribution due to the recent human-mediated transoceanic dispersal of *Angomonas* by *Chrysomya.* This study provides the most comprehensive data gathered so far on the genetic repertoires of a genus of trypanosomatids found in flies from a wide geographical range.

## Introduction

The family Trypanosomatidae (Euglenozoa, Kinetoplastea) is comprised of flagellates that are parasites of vertebrates, plants, and insects. Insects can host trypanosomatid parasites of their own, but they also allow for the cyclical development and transmission of trypanosomatids to plants and vertebrates (Wallace, 1966, 1979; Camargo, 1999; Maslov et al., 2013; Lukeš et al., 2014). Four genera of insect trypanosomatids harbor bacterial endosymbionts: *Angomonas, Strigomonas* (Teixeira et al., 2011), *Kentomonas* (Votýpka et al., 2014), and *Novymonas* (Kostygov et al., 2016). The first three genera constitute the subfamily Strigomonadinae (Votýpka et al., 2014), henceforward referred as symbiont-harboring trypanosomatids (SHTs). The obligate endosymbionts of *Angomonas* species belong to the Betaproteobacteria (Teixeira et al., 2011; Alves et al., 2013a). They are known as trypanosomatid proteobacterial endosymbionts (TPEs), and include two *Candidatus* species, *Ca*. Kinetoplastibacterium desouzai, and *Ca*. K. crithidii (Teixeira et al., 2011).

Trypanosomatid proteobacterial endosymbionts are a model study for symbiosis evolution, they live in the trypanosomatid cytoplasm, replicate synchronically with their hosts, re-shape cell structures, and are vertically transmitted (Freymuller and Camargo, 1981; Motta et al., 2010; Morales et al., 2016). The symbiont-trypanosomatid relationship is characterized by mutually dependent due to reciprocal advantages of their metabolic interactions. Symbionts cannot grow outside of the host trypanosomatid reflecting the limitations imposed by genome streamlining resulting from relaxed selection on genes that are superfluous in the intracellular environment. Nevertheless, the gene arsenal of the symbionts renders their trypanosomatid hosts autotrophic for hemin, essential amino acids and vitamins (Alves et al., 2011, 2013a,b; Klein et al., 2013; Motta et al., 2013). Antibiotic selective elimination of symbionts impairs the metabolic capabilities and growth of the trypanosomatid partner, rendering it unable to colonize insects (Roitman and Camargo, 1985; Catta-Preta et al., 2013). The interdependent metabolic association between hosts and symbionts reflects an ancient co-evolutionary history of the SHTs, as suggested by codivergence supported by congruence between SHT and TPE phylogenies (Teixeira et al., 2011).

*Angomonas* comprises just three species: *A. deanei, A. desouzai*, and *A. ambiguus* (Mundim et al., 1974; Fiorini et al., 1989; Teixeira et al., 2011). Despite being one of the most studied insect trypanosomatids, the single isolate of *A. deanei* analyzed was obtained more than 30 years ago (Mundim et al., 1974). In a previous study, a small sampling of blowflies of *Lucilia* and *Chrysomya* captured in different Brazilian biomes was found to harbor *A. deanei*, *A. desouzai*, and the new species *A. ambiguus* (Teixeira et al., 2011).

Calliphorids (blowflies) currently existing in the Neotropical region are either native or invading flies (McDonagh and Stevens, 2011). Until the 1960s, the most abundant calliphorids in Brazil were *Lucilia eximia* and screwworm flies of *Cochliomyia*, in addition to the cosmopolitan *Lucilia sericata* and *Lucilia cuprina* (Aubertin, 1933; Mello, 1961). Most species of *Lucilia* are limited to determined continents while very few are cosmopolitan such as *L. sericata* (Williams et al., 2016). C*ochliomyia* spp. are endemic to the Americas and two species are common in South America: *Cochliomyia hominivorax* that was eradicated from North and Central America and *Cochliomyia macellaria* dispersed from Argentina to Canada (Yusseff-Vanegas and Agnarsson, 2016). In the 1970s, blowflies of *Chrysomya* arrived in Brazil from Africa (Imbiriba et al., 1978; Guimarães et al., 1978), and shortly dispersed throughout the Latin America reaching North America a few years later (Baumgartner and Greenberg, 1984). Invading blowflies may have transmitted trypanosomatids from their native range to flies of the invaded areas (parasite spillover), and may have become infected with trypanosomatids that already existed in the newly occupied areas (parasite spillback) (Kelly et al., 2009; Wells et al., 2015).

Previous finding of native *Lucilia* and African *Chrysomya* captured in Brazil infected with *Angomonas* spp. (Teixeira et al., 2011), and systematic surveys we have carried out of insect trypanosomatids from wide host species and geographical ranges suggested that Calliphoridae are common hosts of *Angomonas* spp. In addition, *Angomonas* spp. was identified in a few flies from other countries (Týč et al., 2013). Our main goals in the present study were to investigate the genetic diversity and host and geographical ranges of *Angomonas*, to verify the existence of any association between the repertoires of *Angomonas* and Neotropical and Afrotropical blowflies, and to assess possible dispersal of *Angomonas* spp. by blowflies. For these purposes, we carried out a broad survey of trypanosomatids in *Chrysomya* and *Lucilia* from the Neotropical and Afrotropical Ecozones and the Latin American *Cochliomyia* using molecular methods sensitive enough to assess interspecific and intra-specific genetic diversity of *Angomonas* spp., and their respective symbionts.

## Materials and Methods

### Sampling of Flies and Identification in Africa, and South and Central America

In the last decade, we have systematically surveyed flies for the presence of trypanosomatids in general by culturing and PCR-screening. We examined 3,900 flies collected manually (mostly from 2007 to 2016) of the families Calliphoridae, Muscidae, Syrphidae, and Sarcophagidae. The Calliphoridae constituted most of the sample collected (2,418 flies, ∼ 62%) and they were the focus of this work. The blowflies examined were classified as: *Chrysomya* (*C. putoria, C. albiceps, C. megacephala*); *Lucilia* (*L. eximia, L. sericata/L. cuprina*) and *Cochliomyia* (*C. hominivorax, C. macellaria*). No molecular attempts were made to distinguish *L. sericata* from *L. cuprina*, which are morphologically very similar, thus specimens collected in Neotropical regions were classified as *L. sericata* following the recommended classification (Williams et al., 2016). Species and geographic origin of all flies examined in the present study are in Supplementary Table 1.

**FIGURE 1:**
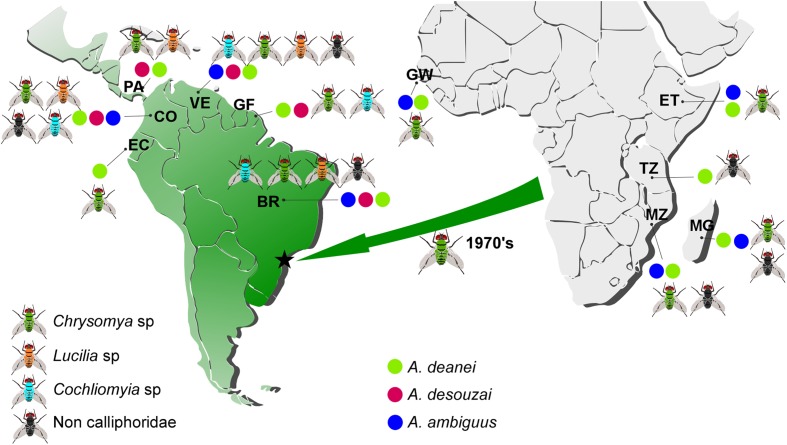
Map indicating the collecting sites of flies and distribution of *Angomonas* species in Afrotropical and Neotropical regions. Blowflies of genera *Chrysomya*, *Lucilia*, and *Cochliomyia* were found infected with *Angomonas deanei*, *A. desouzai*, and *A. ambiguus*. The green arrow indicates the radiation of the African *Chrysomya*, and the green gradient its dissemination from Southern Brazil to across South and Central America. The black star indicates the site of the first reported *Chrysomya* in the Neotropic. BR, Brazil; VE, Venezuela; CO, Colombia; EC, Ecuador; GF, French Guiana; PA, Panama; GW, Guinea Bissau; ET, Ethiopia; TZ, Tanzania; MZ, Mozambique; MG, Madagascar.

In the Afrotropical region, flies were collected in Guinea-Bissau (cities of Bissau and Buba); Mozambique (Beira, Tete, Chupanga, Maputo, Marromeu, and Gorongosa); Madagascar (Andasibe, Antsiranana, Antananarivo, and Moramanga); South Africa (Pretoria); Tanzania (Dar es Salaam and Zanzibar); and Ethiopia (Addis Ababa, Arba Minch, Harar, Hawassa and Lalibela [2,500 m]). In Central America, flies were collected in Panamá city. In South America, sampling of flies extended from northern to southern regions. In Venezuela, flies were collected in Merida and Laguna of Mucubaji (3,500 m) in the Andean Mountains, and in the wetlands of Santa Barbara. In Colombia, flies were collected in Ibagué and near villages between the Central and Oriental Andes Cordilleras. In Ecuador, flies were collected in Ingapirca and Quito (2,850 m). In French Guyana, calliphorids were collected in Cayenne. In Brazil, flies were collected in domestic and sylvatic environments from northern to southern regions across the biomes of the Amazonia, Pantanal, Atlantic Forest, Cerrado, and Caatinga. The locations where flies were collected are shown in **Figure 1** and **Table 1** (detailed in Supplementary Table 1). Blowflies were identified using morphological parameters, and African and American specimens representative of different species from each country were also identified by cytochrome c oxidase 1 (COI) barcoding (Marinho et al., 2012).

**Table 1:** Identification of *Angomonas* species and genotypes in cultures^a^ and guts of blowflies^b^ and other flies^c^ based on V7V8 SSU rRNA plus gGAPDH and/or symbiont-GAPDH sequences.

Species genotype	Blowflies	Other flies	Total
*A. deanei*	141 (75%)	43 (95%)	184 (79%)
Dea1	59	14	73
Dea2	2	14	16
Dea3	76	6	82
Dea4	4	9	13
*A. desouzai*	36 (19%)	2 (5%)	38 (16%)
*A. ambiguus*	12 (6%)	0 (0%)	12 (5%)
total	189 (100%)	45 (100%)	234 (100%)


*^a^175 sequences obtained from 175 cultures of trypanosomatids isolated from species of Lucilia, Chrysomya, and Cochliomyia.*

**FIGURE 2:**
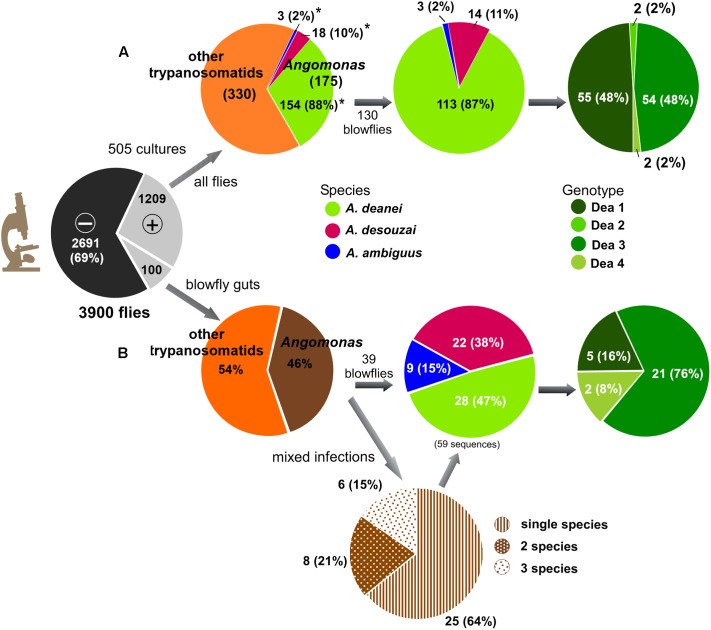
Genetic diversity of *Angomonas* in flies collected in Africa and Latin America. Surveys of trypanosomatids carried out by culturing on 3,900 flies followed by molecular analyses unveiled *A. deanei*, *A. desouzai*, and *A. ambiguus*, and four genotypes (Dea1–Dea4) of *A. deanei* in: **(A)** 505 cultures obtained from 1,209 fly guts microscopically positive (+) for trypanosomatids: 175 cultures were identified as *Angomonas* spp. by V7V8 SSU rRNA barcoding, and 130 cultures were further characterized using GAPDH sequences; **(B)** 100 blowfly microscopically positive guts surveyed by PCR-screening based on symbiont-GAPDH sequences. Sou, *A. desouzai*; Amb, *A. ambiguus*; Dea1–Dea4, *A. deanei* genotypes.

*^b^59 sequences obtained from 39 blowflies (mixed infections showed in **Figure 2B**).*

*^c^Muscidae, Syrphidae, and Sarcophagidae.*

*Detailed data regarding blowfly species and geographical origin are shown in Supplementary Table 1.*

### Surveys of Flies Carrying Trypanosomatids and the Selection of *Angomonas* spp.

The gut contents of dissected flies were microscopically examined for trypanosomatids. Samples that were microscopically positive for trypanosomatids were inoculated in culture tubes containing TC100 medium (=Grace’s medium) containing 20 μg/ml of gentamicin, and incubated at 25°C. Positive cultures cleared of bacteria and fungi were cloned and re-cloned using agar plates (TC100 medium with 2% agar base) (Sbravate et al., 1989; Borghesan et al., 2013). Then, well-separated colonies were transferred to liquid medium, and flagellates checked regarding clonality by sequencing of V7V8 SSU rRNA and/or gGAPDH genes. Cryopreserved cultures, ethanol preserved gut samples, and whole flies were deposited in the Trypanosomatid Culture Collection of the University of São Paulo (TCC-USP). With the aim of detecting *Angomonas*, DNA from guts of blowflies that were positive for trypanosomatids were submitted to V7V8 SSU rRNA barcoding using primers and PCR conditions described previously (Borghesan et al., 2013), and sequences obtained employed for *Angomonas* identification (**Figure 2**).

### Detection of Trypanosomatid Proteobacterial Endosymbionts of *Angomonas*

To specifically detect the TPEs of *Angomonas* spp. using DNA obtained from fly guts, we designed primers based on GAPDH sequences retrieved from published genomes of *A. deanei* and *A. desouzai* symbionts: GAPDH.ENDO.F (5′AAGAGCTCATTATGAAGGTGG3′) and GAPDH.ENDO.R (5′TGGAATCATAYTCATGGTTGC3′). PCR reaction mixtures (25 μl) contained 50–100 ng of DNA, 100 ng of each primer, 200 μM of each dNTP, 1.5 mM of MgCl**_2,_** and 2.5 U of Taq DNA polymerase. PCR reactions were carried out with 30 cycles of 3 min at 94°C, 2 min at 58°C, and 2 min at 72°C, and a final extension for 10 min at 72°C. PCR-screening based on GAPDH sequences of TPEs were used to select fly samples containing *Angomonas* for further analyses. All TPE sequences obtained herein were aligned with GenBank available sequences and submitted to phylogenetic inferences (parsimony, as described below). This approach allows identification of known and novel TPEs of *Angomonas*.

### Barcoding and Phylogeny of *Angomonas* Based on V7V8 SSU rRNA and gGAPDH Sequences

PCR-sequencing of the variable V7V8 SSU rRNA and gGAPDH from *Angomonas* cultures and fly gut samples were performed as described previously (Borghesan et al., 2013). Sequences obtained were screened for chimera using the RDP4 package (Martin et al., 2015), and aligned with sequences from species of several genera representative of the main clades of Trypanosomatidae using ClustalX. Three alignments, were used for phylogenetic inferences. Alignment A1 comprised V7V8 SSU rRNA gene (810 characters) from SHTs, and was submitted to parsimony analysis. A2 included concatenated V7V8 SSU rRNA and gGAPDH sequences (1,957 characters) from representatives of all *Angomonas* spp., other species of SHTs, and various trypanosomatid genera; analyses were performed by maximum likelihood (ML) and Bayesian inference (BI) methods. A3 contained gGAPDH sequences (864 characters) representative of all species and genotypes of *Angomonas* using species of the genus *Strigomonas* as outgroups, and submitted to parsimony. Parsimony and bootstrap analyses were performed using PAUP^∗^ version 4.0b10 software (Swofford, 2002) with 500 random sequence replicates followed by branch swapping (RAS-TBR). ML analyses were performed using the general time reversible (GTR)-gamma-I model in RAxML v.7.0.4 (Stamatakis, 2006) with 1,000 maximum parsimony-starting trees. Model parameters were estimated using the randomized accelerated maximum likelihood (RAxML) model over the duration of the tree search. Bootstrap support was calculated by including 1,000 replicates in the RAxML model using the rapid bootstrapping algorithm, and maximum parsimony as starting trees. Bayesian analysis was performed using MrBayes v3.1.2, the tree searches employed GTR-gamma-I, and the first 25% of the trees from 1,000,000 generations were discarded as burn-in. Default values were used for the remaining parameters. Sequence divergences were calculated using distance matrices (uncorrected *p*-distance) that were constructed using the program PONTOS (^1^). Sequences of V7V8 SSU rRNA and gGAPDH genes representative of all *Angomonas* species and genotypes, from different flies and geographical origin, determined in the present study were deposited in GenBank. The accession numbers of these sequences, and those from previously sequenced SHTs are shown in Supplementary Table 2.

To evaluate whether SHTs and TPEs have undergone parallel diversification, the significance of topological congruence was assessed using the program TreeMap 3.0β, a pairwise distance correlation test of topological congruence that allowed for graphic representation (tanglegrams) of coevolutionary, host-switching, and other events (Charleston and Robertson, 2002). TreeMap uses reconciled trees to compute the fit between the host and parasite phylogenies, and includes a statistical testing that generated random trees to assess whether co-divergence is significantly higher than chance alone.

### Polymorphism Analysis of Internal Transcribed Spacer-1 of rDNA from *Angomonas* spp.

Sequences of PCR-amplified internal transcribed spacer-1 (ITS1) of rDNA were determined using primers and PCR reaction described previously (Borghesan et al., 2013). Sequences obtained in the present study were aligned with those described previously for *Angomonas* spp. (from GenBank), and employed for phylogenetic analysis using parsimony as described above. Secondary structures of ITS rDNA were inferred using the Unafold package (thermodynamically predicted models) with the energy minimization of paired and unpaired regions at 37°C folding temperatures (Markham and Zuker, 2008), as described previously for trypanosomatids of the genus *Herpetomonas* (Borghesan et al., 2013). The secondary structures were visualized by using the VARNA program (Darty et al., 2009). All ITS1 rDNA sequences employed in this study are deposited in GenBank, and the accession numbers are shown in Supplementary Table 2.

### Statistical Analyses

Analyses of genetic structures of *Angomonas* populations were tested using AMOVA implemented in Arlequin v. 3.5.1.2 (Excoffier and Lischer, 2010), and the significance was calculated using 20,000 permutations. Statistical analyses were done using Epi Info public domain site. The correlation between the geographic and genetic distance matrices was investigated with the Mantel test version 2.0 (Liedloff, 1999), with 1,000 iterations for the calculation of the random distribution. Fisher’s Test, a standard procedure for measuring the statistical association between two discrete variables, were used to assess the significance of associations between *Angomonas* species/genotypes and geographical origin or host species.

## Results

### Identification of *Angomonas* spp. from Cultures by V7V8 SSU rRNA Barcoding

Microscopy revealed the presence of trypanosomatids in 1,209 out of 3,900 flies from the families Calliphoridae, Sarcophagidae, Muscidae, and Syrphidae, yielding an estimated overall prevalence of 31%. Gut contents of all microscopically positive flies were inoculated in culture, and 505 cultures of different genera of trypanosomatids were obtained, yielding a 42% rate of culturing success. In the last 15 years, V7V8 SSU rRNA barcoding of trypanosomatids from a range of fly families has been systematically carried out in our laboratory. Barcoding of 505 cultures of fly trypanosomatids allowed to identify 175 (34%) cultures harboring *Angomonas* spp.: 154 *A. deanei* (88%), 18 (10%) *A. desouzai*, and 3 (2%) *A. ambiguus*. Barcoding of trypanosomatids from non-cloned cultures and guts from blowflies revealed the coexistence of *Angomonas* spp., and other species that require further phylogenetic analyses. Among 130 cloned cultures of *Angomonas* from blowflies, 113 (87%) were identified as *A. deanei*, 14 (11%) as *A. desouzai*, and 3 (2%) as *A. ambiguus* (**Figure 2A**).

**FIGURE 3:**
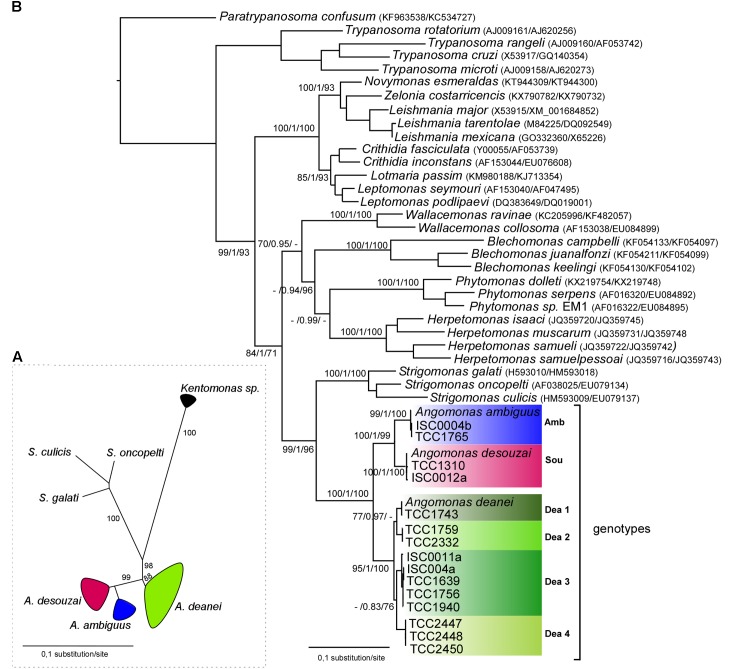
Phylogenetic analysis of *Angomonas* species and genotypes. **(A)** Dendrogram (ML) of V7V8 SSU rRNA sequences (barcodes) from 175 (174 from blowflies) cultures of *A. deanei* (*Dea*), *A. desouzai* (Amb), and *A. ambiguus* (Sou), and species of the other genera of symbiont harboring trypanosomatids: *Strigomonas culicis*, *Strigomonas oncopelti*, *Strigomonas galati*, and *Kentomonas sorsogonicus.* Numbers at the nodes are ML support values (>50%) from 500 replicates. **(B)** Positioning of representatives of each the three *Angomonas* species and four genotypes of *A. deanei* (Dea1–Dea4) were showed in the phylogenetic tree of Trypanosomatidae based on concatenated gGAPDH and V7V8 SSU rRNA gene sequences, inferred by maximum likelihood (–Ln = –16564.401081) and Bayesian analyses. Numbers at the nodes correspond, respectively, to ML/BI/P support values (>50%) from 500 replicates. GenBank accession numbers of V7V8 SSU rRNA and gGAPDH sequences are shown in Supplementary Table 2.

Phylogenetic analysis of V7V8 SSU rRNA sequences from cultured *Angomonas* spp. showed three strongly supported clades corresponding to the three species, *A. deanei*, *A. desouzai*, and *A. ambiguus* (**Figure 3A**). Phylogeny including all know species of the different genera of SHT supported three separate clades, *Angomonas, Strigomonas*, and *Kentomonas* (**Figure 3A**), which together form the subfamily Strigomonadinae (Votýpka et al., 2014). V7V8 SSU rRNA sequences failed to disclose infraspecific diversity within *Angomonas* spp. Most V7V8 SSU rRNA sequences employed in this analysis were determined in the present study, and sequences representative of each species/genotypes were deposited in Genbank (Supplementary Table 2).

### Identification of *Angomonas* spp. from Blowfly Guts by PCR-Amplification of Symbiont GAPDH Sequences

Comparison of symbiont-GAPDH sequences from all *Angomonas* spp. discovered relevant polymorphisms among sequences obtained for 113 cultures of *A. deanei*, whereas sequences from *A. desouzai* and *A. ambiguus* were more homogeneous. Analysis of symbiont-GAPDH sequences allowed to identify four genotypes of *A. deanei*: Dea1, 55 cultures (48%); Dea3, 54 cultures (48%); Dea2 and Dea4, two cultures each (2%) (**Figure 2A**).

PCR-amplification of symbiont-GAPDH sequences from gut content of 100 blowflies revealed 46 flies (46%) harboring *Angomonas* spp. Further analysis of 39 flies revealed infections with a single species in 25 (64%) flies, and mixed infections with two and three species in 8 (21%) and 6 (15%) flies, respectively, yielding a total of 59 symbiont-GAPDH sequences. These sequences showed a predominance of *A. deanei* (28 sequences, 47%) followed by *A. desouzai* (22 sequences, 38%), and *A. ambiguus* (9 sequences, 15%) (**Figure 2B**). The results reveal that *A. ambiguus* and *A. desouzai* have a higher prevalence (7 and 3.5 times, respectively) in blowfly gut samples than in cultures (*p* < 0.01), confirming experimental observation that culturing favors the growth of *A. deanei* over *A. desouzai* and *A. ambiguus*. In addition, the genotype Dea3 (76%) prevails over Dea1 (16%) in blowfly guts, thus differing from cultures showing 48% prevalence rates for both Dea1 and Dea3 genotypes. Notably, not a single species or even genotype of *Angomonas* distinct from those found in cultures were unveiled by direct examination of blowfly gut samples.

Altogether, the identification of *Angomonas* spp. among trypanosomatids of cultures and blowfly guts by the combination of SSU rRNA barcoding plus symbiont-GAPDH genotyping revealed *A. deanei* in 184 flies (141 blowflies), *A. desouzai* in 38 (36 blowflies), and *A. ambiguus* in 12 blowflies (**Figures 2A,B** and **Table 1**).

### Phylogenetic Relationships and Genetic Repertoire of *Angomonas* Inferred by SSU rRNA and gGAPDH Phylogenetic Analyses

Analyses of gGAPDH sequences of *Angomonas* spp. corroborated species identification based on V7V8 SSU rRNA, and genotyping based on symbiont-GAPDH sequences. Phylogenetic analysis using V7V8 SSU rRNA and gGAPDH sequences strongly supported three clades corresponding to the three species of *Angomonas* as showed in the phylogenetic tree inferred using isolates representative of each species/genotypes (**Figures 3A,B**). In addition, this analysis strongly support the partition of *A. deanei* in two main lineages, with infra-lineage diversity (moderately supported) suggesting the existence of four genotypes of this species (**Figure 3B**).

**FIGURE 4:**
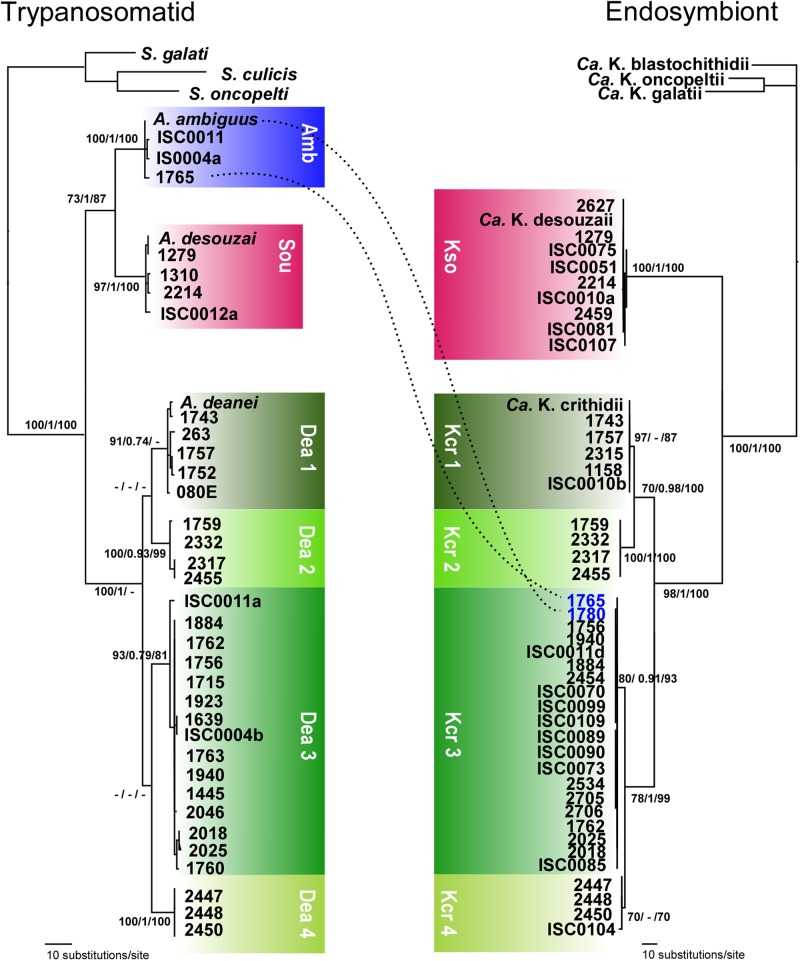
Congruence between *Angomonas* spp. and endosymbiont phylogenies. Parity analysis between phylogenies inferred using sequences of gGAPDH from the trypanosomatid hosts and GAPDH from their respective symbionts. Host and corresponding symbiont genotypes are represented by matching colors. The dotted line indicates the presence of Kcr3 symbiont of *A. deanei* in *A. ambiguus.* Amb, *A. ambiguus;* Sou, *A. desouzai*; Dea1–Dea4, *A. deanei* genotypes. Kso, *Candidatus* Kinetoplastibacterium desouzaii symbiont of *A. desouzai;* Kcr1–Kcr4, genotypes of *Candidatus* Kinetoplastibacterium crithidii symbiont of *A. deanei*. Numbers at the nodes correspond, respectively, to P/ML/BI support values (>50%) from 500 replicates. GenBank accession numbers of sequences employed in this analysis are shown in Supplementary Table 2.

**FIGURE 5:**
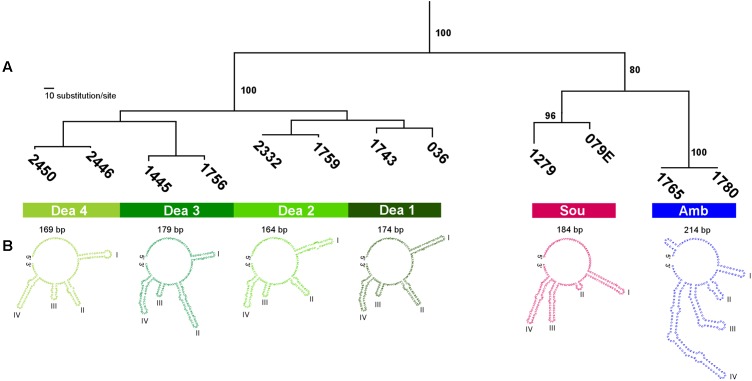
Analyses of ITS1 rDNA sequences from *Angomonas* species and genotypes. **(A)** Dendrogram (parsimony) of ITS1 rDNA sequences from *A. ambiguus*, *A. desouzai*, and genotypes (Dea1–Dea4) of *A. deanei*. The numbers at the node correspond to bootstrap values (>50%) from 500 replicates. **(B)** Predicted secondary structures of ITS1 rDNA sequences from *Angomonas* species and genotypes showing an open loop and four main helices that varied in length according to species and main genotypes of *A. deanei*. The estimated values of thermodynamic energy of modelled structures (ΔG Kcal/mol) for each species were: *A. deanei* (–54.4), *A. desouzai* (–69), and *A. ambiguus* (–83.3). Sou, *A. desouzai*; Amb, *A. ambiguus*; Dea1–Dea4, *A. deanei* genotypes. GenBank accession numbers of ITS1 rDNA sequences included in this analysis are shown in Supplementary Table 2.

In contrast with highly conserved SSU rRNA sequences, the more polymorphic gGAPDH sequences from isolates of *A. deanei* showed relevant sequence divergences supporting the four (Dea1–Dea4) genotypes of this species previously uncovered by comparison of symbiont-GAPDH sequences. Among the 113 cultures of *A. deanei* examined, 55 (48%) were identified as Dea1, 54 (48%) as Dea 3, 2 (2%) as Dea 2, and 2 (2%) as Dea4 (**Figures 2**, **3**). Phylogenetic relationships based on gGAPDH sequences of a larger number of isolates of *A. deanei* strongly supported the Dea1-Dea4 genotypes (**Figure 4**). In addition, the four genotypes of *A. deanei* were strongly supported in the analyses restricted to *Angomonas* based on ITS rDNA (**Figure 5**),

Genotype Dea1 includes the holotype of *A. deanei*, TCC 036E (Teixeira et al., 2011). There were no gGAPDH divergences greater than 0.1% among isolates of the same genotype of *A. deanei.* The gGAPDH sequence divergences among the four Dea genotypes ranged from 2.0 to 3.0%. For comparison, gGAPDH divergences of *A. deanei* from *A. desouzai* and *A. ambiguus* were about 7%, whereas divergences between *A. desouzai* and *A. ambiguus* were 4.0%. New isolates of *A. desouzai* and *A. ambiguus* diverged less than 1.0% from the respective holotypes, thus lacking relevant polymorphisms that would justify their separation in infraspecific genotypes.

### Inter- and Intra-specific Diversity of *Angomonas* spp. Unveiled by ITS1 rDNA Sequences

Aiming at improving the assessment on infraspecific genetic diversity within the species of SHTs, we determined sequences of the highly polymorphic ITS rDNA sequences (Teixeira et al., 2011). ITS rDNA phylogenetic analysis confirmed the clustering of sequences according to species and genotypes (**Figure 5A**). Extensive polymorphisms, with divergences of ∼ 50% in ITS rDNA sequences, separate the cluster containing *A. deanei* genotypes from those formed by *A. desouzai* and *A. ambiguus* (which diverge ∼ 35% from each other), and corroborated the high genetic diversity among *A. deanei* genotypes: ∼ 12% between Dea1and Dea2, and ∼20% between Dea1 and Dea3/Dea4. Sequence divergences within a given genotype were small, varying from 1.0% (within Dea3) to 6.0% (within Dea1). The maximum ITS rDNA sequence divergence among clones of a given isolate was 2.0%. Despite relevant polymorphisms among species and genotypes, the relationships inferred using ITS rDNA sequences (**Figure 5**) corroborated V7V8 SSU rRNA + gGAPDH (**Figure 3B**) and gGAPDH (**Figure 4A**) inferred phylogenetic relationships.

*Angomonas* spp. exhibited the same general features of ITS rDNA secondary structure of trypanosomatids in general, consisting of an open loop with four main helices. The putative secondary structures exhibited four helices that varied in length, shape and position, which allowed for the species of *Angomonas* to be clearly distinguished. The structures of the most divergent Dea1 and Dea3 genotypes were distinguishable, while Dea1 and Dea 2 shared very similar structures (**Figure 5B**). In every case, isolates of the same genotype yielded identical overall structure, which added support to the consistency of secondary structures as one valuable taxonomic parameter to distinguish species and even genotypes of trypanosomatids (Borghesan et al., 2013).

### Parallel Evolution of Symbionts and Their *Angomonas* Hosts Supported by Congruent Phylogenies

Accompanying the genetic heterogeneity of gGAPDH sequences of *Angomonas* spp. hosts (**Figure 4A**), GAPDH sequences of *Angomonas* symbionts also exhibited marked heterogeneity. In the inferred phylogenies, sequences of the symbionts of *A. deanei* were split into two main clusters, each one comprising two well-supported genotypes (three genotypes were herein reported for the first time). We refer to these four genotypes of *Candidatus* Kinetoplastibacterium crithidii as Kcr1, Kcr2, Kcr3, and Kcr4 (**Figure 4B**). The Dea clusters are congruent with the Kcr clusters as supported by ML, P, and BI analyses: Kcr1 associated exclusively with Dea1, Kcr2 with Dea2, Kcr3 with Dea3, and Kcr4 with Dea4. Similarly, isolates of *A. desouzai* paired only with *Ca*. K. desouzaii. The only exception was the lack of congruence between *A. ambiguus* and its symbiont; this species harbors the symbiont Kcr3 of *A. deanei* Dea3 (**Figures 4A,B**).

**FIGURE 6:**
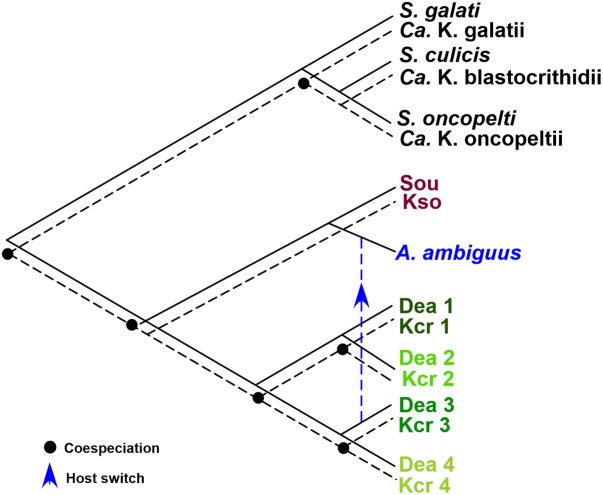
Tanglegram of coevolutionary patterns between *Angomonas* hosts (SHTs) and symbionts (TPEs), strongly supporting (*p* < 0.002) codivergence between species/genotypes of SHTs and species/genotypes of TPEs. The only exception was a single host switch between *A. ambiguus* and the symbiont Kcri3 of *A. deanei* Dea3. Tanglegram generated by TreeMap using the MP topologies inferred with gGAPDH sequences from SHTs and GAPDH from TPEs (showed in **Figure 5**).

To assess whether SHTs and TPEs have undergone parallel diversification, the significance of topological congruence between the phylogenies of the SHT and TPE species and genotypes was assessed by TreeMap. The tanglegram inferred by TreeMap strongly supported (*p* < 0.002) codivergence events between SHTs and TPEs, and a single event of host switch represented by *A. ambiguus* carrying the symbiont Kcri3 of *A. deanei* Dea3 (**Figure 6**).

### Tripartite Association of *Angomonas*, Endosymbionts and Calliphorids throughout Their Geographic Ranges

**FIGURE 7:**
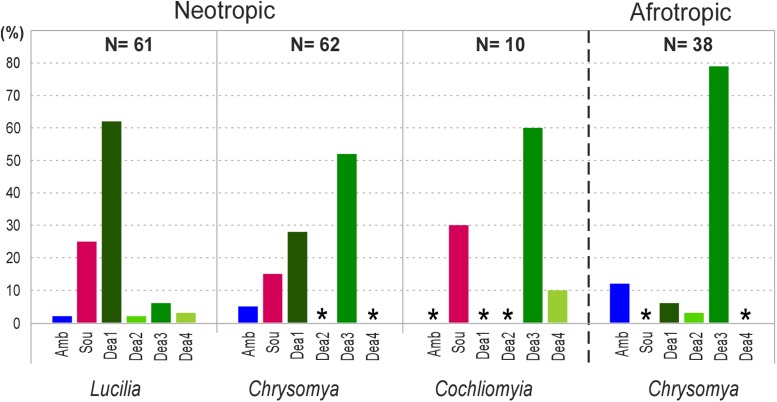
Infection rates and geographical distribution of *Angomonas* species and genotypes in blowflies from the Afrotropic and Neotropic. *A. ambiguus* (Amb), *A. desouzai* (Sou), and *A. deanei* (Dea1–Dea4 genotypes) detected in calliphorids of the genera *Chrysomya*, *Lucilia*, and *Cochliomyia. N* = number of infected blowflies. The asterisk indicates absence. *Angomonas* was not detected in *Lucilia* from the Afrotropic. Detailed data from each fly genera are shown in **Table 1**, and result obtained from each fly culture and gut sample are shown in Supplementary Table 1.

Keeping in mind that *Cochliomyia* spp. and *Lucilia eximia* are New World flies and that, until the 1970’s, *Chrysomya* was absent in the Americas, we scored the presence of *Angomonas* genotypes, and their association with these three genera of Calliphoridae, in the Neotropical and Afrotropical realms (**Figures 1**, **7**). *A. deanei* predominated in all examined genera of blowflies, from Neotropic and Afrotropic, regardless whether the trypanosomatids were analyzed from cultures or directly from fly guts. *C. putoria* and *C. megacephala* as well as in *L. sericata* and *L. eximia* were the commonest hosts of *A. deanei. A. desouzai* was identified exclusively in the Neotropical blowflies of the three calliphorid genera examined; however, most isolates of this species were detected in *L. eximia* followed by *C. putoria.* The absence of *A. desouzai* in *Chrysomya* in Africa contrasts with very high significance (*p* < 0.01) with its presence in the introduced *Chrysomya* spp. *A. ambiguus* was found in the Afrotropic and Neotropic, mainly in *C. putoria* and sporadically in *C. megacephala, C. albiceps*, and *L. eximia* from the New World (**Figures 1**, **7**). Interestingly, this species was not detected in *Cochliomyia*, even in searches carried out directly by PCR using DNA from fly guts to avoid favored culturing of other species. To date, *C. hominivorax* and *C. macellaria* were found infected with *A. deanei* and *A. desouzai* (**Figures 2B**, **7** and **Table 1**).

Regarding genotypes of *A. deanei*, Dea3 was predominant in *Chrysomya* spp. from the Afrotropic and Neotropic, collected either in native or invaded regions (*p* < 0.01). Notably, this genotype was also common in *Cochliomyia.* In contrast, Dea1 was the predominant genotype infecting South American *Lucilia*, either the Neotropical *L. eximia* or exotic *L. cuprina*/*L. sericata.* Compared to Dea3, Dea1 was rarer in African *Chrysomya* spp., whereas it is worth noting the presence of Dea2 exclusively in African *Chrysomya* (**Figure 7**). The repertoire of *A. deanei* uncovered in *C. hominivorax* and *C. macellaria* collected in Brazil, Colombia, Venezuela and French Guiana revealed *A. deanei* of the genotypes Dea3 and Dea4 (**Figures 1**, **7**). In addition, these flies were the major hosts of *A. desouzai*, suggesting (*p* < 0.01) that this species may be endemic to the New Word. Altogether, our data revealed a distinct repertoire of *Angomonas* species and genotypes for each calliphorid genus examined (**Figure 7**). Apparently, Dea1 and Dea3 genotypes have preferences for *Chrysomya* and *Lucilia*, respectively, with very high significance estimated by Fisher’s Test (*p* < 0.01).

### Lack of Genetic Distances between African and American Populations of *Angomonas* spp.

Despite the widespread distribution of *A. deanei* and *A. ambiguus*, divergences of gGAPDH sequences of isolates from distant areas in South America and Africa were always smaller than 1.0%. Similar results were obtained for isolates of *A. desouzai* from distant collecting sites of South America. We used Mantel’s test to assess the correlation between geographic and genetic distances among the main genotypes Dea1 and Dea3 that infect blowflies in the Afrotropic and Neotropic. We found no significant correlation (*r* = –0.0353) between the two matrices, with a standard normal variate (*g*) value of –0.4359 for our dataset (for a significance of 0.05, the critical value is 1.645). Overall, 34.6% of the random permutations exhibited a better correlation between matrices than that of collected data. Thus, no correlation was found between geographic and genetic distances of Dea1 and Dea3 genotypes from Africa and South America what clearly indicates the recentness of their geographical separation.

We also analyzed the genetic structure of *A. deanei* genotypes by comparing gGAPDH sequences from the Afrotropical and Neotropical genotypes Dea1 and Dea3. Using the AMOVA test, results revealed a diversity of 2.4% between the African and South American genotypes, and 97.4% in each population, which supports the lack of spatial genetic structure for both Neotropical and Afrotropical parasites (ф_ST_ 0.024, *p* < 0.003). Regarding the endosymbiont, the analysis of Kcr1 and Kcr3 TPEs confirmed the somewhat higher degree of genetic structure of symbionts. Divergence between gGAPDH sequences amounted to 6.1% between African and South American TPEs, with ∼ 85% divergence within each population (ф_ST_ 0.15).

## Discussion

Here, we provided the most comprehensive data gathered so far regarding the genetic diversity, and ranges of host species and geographical distribution of a genus of trypanosomatids exclusive of insects, mainly flies. The results demonstrated that regardless of their hosts and geographic origin, the populations of *Angomonas* consisted of species and genotypes that clustered together in three strongly supported phylogenetic lineages. Each lineage corresponds to one of three species, *A. deanei* (four genotypes), *A. desouzai*, and *A. ambiguus.* There is a consistent congruence between the phylogenies of these flagellates and their respective symbionts both at species and genotype levels. The only exception is *A. ambiguus* that harbors the symbiont of the Dea3 genotype of *A. deanei*, confirming the ambiguity of *A. ambiguus* as previously demonstrated based on analysis of TPE ITS rDNA and 16S sequences (Teixeira et al., 2011)

It is not known when the *Angomonas–*symbiont association was first established in geologic times. Provisional estimates (Du et al., 1994) suggested that a single event of symbiont acquisition by the SHT ancestor may have occurred by the end of the Cretaceous. However, phylogenetic analyses supported that all TPEs have a common Betaproteobacteria ancestor (Alves et al., 2011, 2013a,b; Klein et al., 2013). The existence of an extinct, therefore, inferable common symbiont ancestor is supported by the overall genomic similarity among *Angomonas* symbionts, and broad genomic synteny (Alves et al., 2013b). Regardless of the timing of the event, SHTs and their symbionts initiated a joint journey of coevolution long ago, which seems to have been influenced by their strong association with flies, and by the cohabitating in the insect guts with a diversity of bacteria. The tightly associated pairs of host–symbiont genotypes suggest coevolution, with long-shared evolutionary histories consistent with interdependence between host and symbiont genomes (Moran, 2006; Moran et al., 2008; Alves et al., 2011, 2013a).

This study brought information about the tripartite association of *Angomonas*, endosymbionts and calliphorids throughout their geographic ranges. *A. deanei* and *A. ambiguus* seem to be cosmopolitan because in addition to Neotropical and Afrotropical realms, they have been also reported in Papua New Guinea, while *A. deanei* has also been found in Europe (Týč et al., 2013). Noticeable was the absence of *A. desouzai* in the Afrotropic, suggesting Neotropical endemicity of this species, and spillback of *A. desouzai* from resident Neotropical hosts to *Chrysomya* in South America. The finding of *A. desouzai* in sarcophagid flies from Ecuador (Týč et al., 2013) corroborated the hypothesis that *A. desouzai* may be endemic to the New Word, but also suggests that flies other than blowflies may be the preferred hosts of this species.

The three-known species of *Angomonas* have been recovered from blowflies, with high prevalence rates, from both hemispheres, from sea level to 3,500 m of altitude in the Andes Cordillera, from 9° north (Panama) to 25° south (South Brazil), and from 47° east (Madagascar) to 77° west (Panama). In Brazil, *Angomonas* spp. Occurred in calliphorid flies from the Amazonian Rainforest, the Pantanal wetlands, Cerrado (savanna), Atlantic Forest, and semiarid Caatinga. Nothing is known about the behavioral and pathogenic implications to blowflies of the highly prevalent *Angonomas* infections. Blowflies of *Chrysomya, Cochliomyia*, and *Lucilia* exhibiting different parasite repertoires were many times collected while feeding together, and it is well known that the spread of insect trypanosomatids occurs by coprophagy (Wallace, 1966; Hupperich et al., 1992). This finding together with different repertoires of *Angomonas* exhibited by each calliphorid genus examined suggested the existence of a certain degree of association between species/genotypes of *Angomonas* and genera/species of Calliphoridae flies.

Geographic distribution of *A. deanei* genotypes (Dea1–Dea4) in *Chrysomya* partially agrees with the general rule that parasite diversity in a determined host is larger in the native area of the invader than in the invaded area (Poulin and Mouillot, 2003; Torchin et al., 2003). Accordingly, a great diversity of genotypes has been uncovered in *Chrysomya* in the Afrotropic (Dea2 was found exclusively in Africa), suggesting a possible bottleneck effect of *A. deanei* genotypes, concordant with the bottleneck of *Chrysomya* when introduced in the New World (Junqueira et al., 2016). However, *Chrysomya* spp. in Afrotropical habitats carried mostly *A. deanei* and sporadically *A. ambiguus*, whereas in the Neotropic, these flies carried the three species of *Angomonas.*

For the first time, the repertoire of trypanosomatids was described in *C. hominivorax* and *C. macellaria*,which are the two most widespread species of this Neotropical genus of blowfly (Yusseff-Vanegas and Agnarsson, 2016). *Cochliomyia* spp. collected for the present study in Brazil, Colombia, Venezuela and French Guiana were found infected with the widespread genotype Dea3, and the rare genotype Dea4. The genotype Dea 3 of *A. deanei* can be associated to *Chrysomya* spp. of both the Neotropic and Afrotropic, whereas Dea1 was present only in South American *Lucilia* spp. It is important to notice the absence in our study of *Angomonas* in *Lucilia* collected in Africa. Apparently, Dea2 and Dea 4 occur preferentially in Muscidae, and their scarcity may be due to our survey focusing on Calliphoridae.

Mitogenomics support the origin of Calliphoridae at the boundary of Oligocene and Miocene, ∼ 22 million years ago (Junqueira et al., 2016), when the ancestors of *Angomonas* were likely parasitizing flies other than calliphorids. There are fossil records showing trypanosomatids associated with insects since the early Cretaceous (Poinar, 2007), but no fossil exists of blowflies harboring these flagellates. Regardless of the dating, results from the present study, and studies currently being done at our laboratories on various fly and hemipteran families, are showing that the extant tripartite association calliphorid–*Angomonas*–symbiont is tightly linked, and that *Angomonas* stands out among the preferred trypanosomatid inquiline of the Calliphoridae. However, whether *Angomonas* spp. prefers the Calliphoridae flies requires additional studies because there are more than one hundred dipteran families, and we examined only four of them.

Although highly prevalent in flies, *A. deanei* was originally recovered from a predator reduviid (Carvalho, 1973). Before that, nothing was published about flies harboring SHTs. However, there are drawings of fly trypanosomatids more than one century old (Roubaud, 1908, 1911a,b) showing opisthomorphs, which are characteristic of SHTs (Teixeira et al., 2011), thus suggesting the presence of *Angomonas* and other SHTs in African calliphorids at least one century ago.

Surveys of blowflies (field surveys and museum collections) revealed the predominance of species of *Lucilia* and *Cochliomyia*, and the absence of *Chrysomya* among Brazilian calliphorids in the 1970s (Mello, 1961; Guimarães et al., 1978, 1979). The Afrotropical *Chrysomya putoria* (=*C. chloropyga*) was for the first time reported in the Neotropic in 1976 in the vicinities of a Southern Brazilian seaport harboring ships from Angola and Mozambique (Imbiriba et al., 1978; Guimarães et al., 1978). Shortly after that, *C. albiceps* and *C. megacephala* were also reported in southeastern Brazil (Guimarães et al., 1979). Then, African blowflies rapidly expanded through the New World (Guimarães et al., 1978, 1979; Richard and Gerrish, 1983; Baumgartner and Greenberg, 1984; Wells, 1991). The growing *Chrysomya* population most likely overwhelmed the population of *Cochliomyia*, which became mainly restricted to more preserved areas of central and northern South America (Batista-da-Silva et al., 2011). Genetic population structure supporting recent introduction of *Chrysomya* blowflies into the New World have been well-documented (Godoy et al., 1997; Rodrigues et al., 2009; McDonagh and Stevens, 2011; Junqueira et al., 2016).

Available data do not permit the establishment of the timeframe and place in which *Angomonas* spp. originated and dispersed to the Neotropic and Afrotropic. After Columbus, any fly hosting *Angomona*s spp. could have been carried in both directions between the Old and New Worlds due to the intensive commerce of people, livestock, and goods (Wells, 1991; McDonagh and Stevens, 2011; Junqueira et al., 2016; Williams et al., 2016). Apparently, *Chrysomya* only became established in the Americas in the last 40 years. The description of *A. deanei* in Brazil before the arrival of *Chrysomya* (Carvalho, 1973; Carvalho and Deane, 1974; Mundim et al., 1974) plus the fact that flies of many families including resident *Lucilia* spp. are hosts of *Angomonas*, may indicate the existence of these flagellates in the American continent before the introduction of *Chrysomya*. Nevertheless, the recent dispersion of the highly prolific African *Chrysomya* spp., and their prompt adaption to diverse environments, particularly anthropogenic habitats, likely played a fundamental role in the rapid dispersion of *Angomonas* spp. throughout South and Central America. The genotypes of *Angomonas* that are present in the Neotropic are indistinguishable from their Afrotropical counterparts, consistent with the recent, and most likely still ongoing, dispersal of *Chrysomya* spp. throughout the world. These findings highlight the current disjunct distribution of *Angomonas* spp., and support its trans-oceanic dispersal (de Queiroz, 2014) through man-mediated spreading of flies harboring *Angomonas*–TPE pairs.

The very comprehensive data gathered in the present study, and results from two previous smaller surveys (Teixeira et al., 2011; Týč et al., 2013), demonstrated that *Angomonas* occurs in the Neotropic, Afrotropic, Palearctic, Indomalaya, and Australasia ecozones. The findings herein reported on the genetic repertoire of *Angomonas* spp. in calliphorid flies in the Neotropic and Afrotropic is the first step toward a broader phylogeography of the tripartite *Angomonas–*symbiont–blowfly association. In this study, we examined just the more prevalent species of three Calliphoridae genera from the Neotropic and Afrotropic. Our findings underscore the need to extend data collection of blowflies, and many other flies from wider geographical range to better understand the repertoire composition of *Angomonas-*TPE species and genotypes, and their dispersal and evolutionary dynamics throughout the world. Notably, SHTs of the genus *Strigomonas* were not detected in blowflies, and to date species of *Kentomonas* were reported only in Sarcophagidae from the Philippines (Votýpka et al., 2014). A better knowledge on the host-species and geographical ranges of these genera is crucial to the understanding of the evolutionary history of the whole Strigomonadinae subfamily.

## Author Contributions

TB, MC, and TM are co-first authors; TB and TM performed the sequencing and phylogenetic analyses; MC was responsible for the isolation and culturing of parasites; OE, VR, FP, JC, NA, and LN coordinated the field works, and help collection, dissection and microscopical examination of flies; EC and MT designed the study, organized the data set, drafted the manuscript, and share co-last authorship. All authors contributed with data analysis, share the responsibility related to the accuracy of the work, revised the manuscript, and approved its final version.

## Conflict of Interest Statement

The authors declare that the research was conducted in the absence of any commercial or financial relationships that could be construed as a potential conflict of interest.

## Footnotes

1 http://sourceforge.net/projects/pontos/
