# Supplementary material for: Genetic Diversity and Phylogenetic Relationships of Coevolving Symbiont-Harboring Insect Trypanosomatids, and Their Neotropical Dispersal by Invader African Blowflies (Calliphoridae)
Source: Front Microbiol. 2018 Feb 7;9:131. doi: 10.3389/fmicb.2018.00131 (PMC5808337; doi:10.3389/fmicb.2018.00131)
Supplement: Supplementary file 1 [file Table_1.PDF]

Supplementary Table 1

Host species and geographical origin (ecozone) of species and genotypes of *Angomonas*

Species	Host origin		Geographical origin		
genotypes	Family	species	ecozone	Country (state)	Year
Angomonas deanei					
**Dea1**					
TCC1503, 1504, 1505	Calliphoridae	*Chrysomya albiceps*	NT	BR (MS)	2007
ISC0010b	Calliphoridae	*Chrysomya megacephala*	NT	VE	2012
TCC2690	Calliphoridae	*Chrysomya megacephala*	NT	VE	2013
TCC2791, 2798, 2799, 2804, 2808, 2820	Calliphoridae	*Chrysomya megacephala*	NT	BR (SP)	2014
TCC263E	Calliphoridae	*Chrysomya putoria*	NT	BR (PR)	1988
TCC1757	Calliphoridae	*Chrysomya putoria*	NT	BR (SP)	1989
ISC0096a	Calliphoridae	*Chrysomya putoria*	NT	BR (MS)	2010
TCC2315	Calliphoridae	*Chrysomya putoria*	AT	MZ	2012
TCC2316	Calliphoridae	*Chrysomya putoria*	AT	MZ	2012
TCC2616	Calliphoridae	*Chrysomya* sp.	NT	PA	2013
TCC2768	Calliphoridae	*Chrysomya* sp.	NT	BR (SP)	2014
TCC2817	Calliphoridae	*Chrysomya* sp.	NT	BR (SP)	2014
TCC2873	Calliphoridae	*Chrysomya* sp.	NT	EC	2015
TCC1743, 1752	Calliphoridae	*Lucilia sericata*	NT	BR (SP)	2008
TCC2466	Calliphoridae	*Lucilia sericata*	NT	BR (TO)	2014
TCC2523	Calliphoridae	*Lucilia sericata*	NT	BR (TO)	2014
TCC2615	Calliphoridae	*Lucilia eximia*	NT	PA	2013
TCC2624	Calliphoridae	*Lucilia eximia*	NT	VE	2013
TCC2628, 2629	Calliphoridae	*Lucilia eximia*	NT	PA	2013
TCC2631, 2636, 2651, 2652	Calliphoridae	*Lucilia eximia*	NT	VE	2013
TCC2663, 2681, 2691	Calliphoridae	*Lucilia eximia*	NT	VE	2013
ISC0013c	Calliphoridae	*Lucilia eximia*	NT	CO	2013
TCC2619	Calliphoridae	*Lucilia* sp.	NT	PA	2013
TCC2762, 2766, 2769, 2771, 2772, 2775, 2777, 2778, 2785, 2788, 2792, 2795, 2796, 2797, 2801, 2802, 2809, 2811, 2813, 2819, 2821, 2824	Calliphoridae	*Lucilia* sp.	NT	BR (SP)	2014
TCC2314	Muscidae	*Musca domestica*	AT	MZ	2012
TCC2491	Muscidae	*Musca* sp.	NT	BR (TO)	2014
TCC2667	Muscidae	ni	NT	VE	2013
TCC2717	Muscidae	ni	NT	BR (TO)•	2014
TCC2776, 2789, 2823	Muscidae	ni	NT	BR (SP)	2014
TCC2653, 2661	Sarcophagidae	ni	NT	VE	2013
TCC2686	Sarcophagidae	ni	NT	VE	2013
TCC080E	Syrphidae	*Ornidia obesa*	NT	BR (MG)	1982
TCC1158	Syrphidae	*Ornidia obesa*	NT	BR (SP)	2006
TCC036E#	Reduviidae	*Zelus leucogrammus*	NT	BR (GO)	1973
**Dea2**					
TCC1759	Calliphoridae	*Chrysomya putoria*	AT	GW	2008
TCC2630	Calliphoridae	*Lucilia eximia*	NT	VE	2013
TCC2332, 2317	Muscidae	*Musca domestica*	AT	MZ	2012
TCC2455	Muscidae	*Musca domestica*	NT	CO	2013
TCC2529	Muscidae	ni	AT	MG	2013
TCC2881, 2882, 2883, 2885, 2886, 2892, 2894, 2897, 2898, 2902	Muscidae	ni	AT	TZ	2015
**Dea3**					
TCC2054, 2070, 2093	Calliphoridae	*Chrysomya albiceps*	NT	BR (MS)	2010
TCC1445	Calliphoridae	*Chrysomya megacephala*	NT	BR (MS)	2007

TCC1742, 1754, 1758, 1884	Calliphoridae	*Chrysomya megacephala*	NT	BR (RO)	2008
TCC1639	Calliphoridae	*Chrysomya magacephala*	AT	MZ	2008
TCC1884	Calliphoridae	*Chrysomya megacephala*	NT	BR (MT)	2009
TCC2046, 2065, 2066, 2067, 2068	Calliphoridae	*Chrysomya megacephala*	NT	BR (MS)	2010
ISC0109	Calliphoridae	*Chrysomya megacephala*	NT	VE	2012
TCC2644	Calliphoridae	*Chrysomya megacephala*	NT	BR (MT)*	2013
TCC2680	Calliphoridae	*Chrysomya megacephala*	NT	VE	2013
TCC2457	Calliphoridae	*Chrysomya megacephala*	NT	CO	2013
TCC2783	Calliphoridae	*Chrysomya megacephala*	NT	BR	2014
TCC1267	Calliphoridae	*Chrysomya putoria*	AT	MZ	2006
TCC1675	Calliphoridae	*Chrysomya putoria*	AT	MZ	2007
TCC1760, 1762, 1763	Calliphoridae	*Chrysomya putoria*	AT	GW	2008
TCC1923	Calliphoridae	*Chrysomya putoria*	AT	MZ	2009
ISC0073	Calliphoridae	*Chrysomya putoria*	AT	MZ	2009
TCC2018, 2025	Calliphoridae	*Chrysomya putoria*	AT	GW	2010
ISC0095, ISC0096b,	Calliphoridae	*Chrysomya putoria*	NT	BR (MS)	2010
ISC0099	Calliphoridae	*Chrysomya putoria*	NT	BR (MS)*	2011
ISC0004a	Calliphoridae	*Chrysomya putoria*	AT	MG	2012
TCC2528, 2542, 2545, 2551, 2561, 2623	Calliphoridae	*Chrysomya putoria*	AT	MG	2013
TCC2531, 2534, 2543, 2552	Calliphoridae	*Chrysomya putoria*	AT	MG	2013
TCC2601	Calliphoridae	*Chrysomya putoria*	AT	MG	2013
ISC0011a	Calliphoridae	*Chrysomya putoria*	NT	CO	2013
ISC0088, ISC0090, ISC0089	Calliphoridae	*Chrysomya putoria*	AT	MG	2013
TCC2705, 2706	Calliphoridae	*Chrysomya putoria*	AT	ET	2014
TCC2944, 2945, 2957, 2958, 2963, 2965	Calliphoridae	*Chrysomya* sp.	NT	GF	2016
ISC0102	Calliphoridae	*Cochliomyia hominivorax*	NT	BR (MS)*	2011
TCC2635	Calliphoridae	*Cochliomyia hominivorax*	NT	VE	2013
TCC1940	Calliphoridae	*Cochliomyia macellaria*	NT	BR (MS)*	2009
ISC0085, ISC0050	Calliphoridae	*Cochliomyia macellaria*	NT	CO	2013
TCC2052	Calliphoridae	*Cochliomyia* sp.	NT	BR (MS)	2010
TCC2946	Calliphoridae	*Cochliomyia* sp.	NT	GF	2016
TCC1447	Calliphoridae	*Lucilia eximia*	NT	BR (MS)	2007
TCC1755, 1756	Calliphoridae	*Lucilia sericata*	NT	BR (RO)	2008
TCC1715	Calliphoridae	*Lucilia* sp.	NT	BR (SP)	1989
ISC0070	Calliphoridae	*Lucilia* sp.	NT	PA	2013
TCC2553, 2554	Muscidae	*Musca domestica*	AT	MG	2013
TCC2606	Muscida	*Musca domestica*	NT	BR (MT)*	2013
TCC1920	Sarcophagidae	ni	AT	MZ	2009
TCC2454	Sarcophagidae	ni	NT	BR (TO)	2013
TCC2313	Gl.	*Glossina* sp.	AT	MZ	2012
**Dea4**					
ISC0104	Calliphoridae	*Cochliomyia hominivorax*	NT	BR (MS)*	2011
TCC2763, 2793	Calliphoridae	*Lucilia eximia*	NT	BR (SP)	2014
TCC2446, 2447, 2448, 2449, 2450, 2453	Muscidae	*Musca domestica*	NT	CO	2013
TCC2645	Muscidae	*Musca domestica*	NT	VE	2013
TCC2893, 2899	Muscidae	ni	AT	TZ	2015
A. desouzai					
TCC1883	Calliphoridae	*Chrysomya megacephala*	NT	BR (MT)	2009
ISC0110	Calliphoridae	*Chrysomya magacephala*	NT	VE	2012
ISC0010a	Calliphoridae	*Chrysomya megacephala*	NT	VE	2012
ISC0096	Calliphoridae	*Chrysomya putoria*	NT	BR (MS)	2010
ISC0012a	Calliphoridae	*Chrysomya putoria*	NT	CO	2013
ISC0105, ISC0107, ISC0108	Calliphoridae	*Chrysomya* sp.	NT	VE	2012
TCC2462	Calliphoridae	*Chrysomya* sp.	NT	BR (TO)	2013
TCC2956	Calliphoridae	*Chrysomya* sp.	NT	GF	2015
ISC0081	Calliphoridae	*Cochliomyia hominivorax*	NT	CO	2013
ISC0050a, ISC0051	Calliphoridae	*Cochliomyia macellaria*	NT	CO	2013
ISC0075, ISC0077, ISC0078	Calliphoridae	*Lucilia sericata*	NT	BR (TO)	2013
TCC1279, 1310, 1311, 1312, 1429, 1453	Calliphoridae	*Lucilia eximia*	NT	BR (MS)	2006/2007
TCC2214	Calliphoridae	*Lucilia eximia*	NT	BR (MS)*	2011
ISC0016b	Calliphoridae	*Lucilia eximia*	NT	VE	2012

TCC2459, 2460	Calliphoridae	*Lucilia eximia*	NT	BR (SP)	2013
TCC2627	Calliphoridae	*Lucilia eximia*	NT	PA	2013
ISC0084	Calliphoridae	*Lucilia eximia*	NT	CO	2013
TCC2626	Calliphoridae	Lucilia sp.	NT	PA	2013
TCC2451	Muscidae	ni	NT	CO	2013
TCC079E#	Syrphidae	*Ornidia obesa*	NT	BR (MG)	1989
A. ambiguus					
TCC1780#	Calliphoridae	*Chrysomya albiceps*	NT	BR (MS)	2007
ISC0010	Calliphoridae	*Chrysomya megacephala*	NT	VE	2012
TCC1765	Calliphoridae	*Chrysomya putoria*	AT	GW	2008
ISC0073	Calliphoridae	*Chrysomya putoria*	AT	MZ	2009
ISC0004b	Calliphoridae	*Chrysomya putoria*	AT	MG	2012
ISC0011	Calliphoridae	*Chrysomya putoria*	NT	CO	2013
TCC2935	Calliphoridae	*Chrysomya* sp.	AT	ET	2016
ISC0138	Calliphoridae	*Chrysomya* sp.	NT	BR (MS)	2010
ISC0013b	Calliphoridae	*Lucilia eximia*	NT	CO	2013

TCC, Trypanosomatid Culture Collection; **ISC**: Insect Sample Collection of the University of São Paulo. BR, Brazil; CO, Colombia; EC, Ecuador; ET, Ethiopia; GF, French Guyana; GW, Guinea Bissau; MG, Madagascar; MZ, Mozambique; PA, Panamá; TZ, Tanzania; VE, Venezuela. Brazilian Biomes (States): **Mata Atlântica**: SP, São Paulo; PR, Paraná; MG, Minas Gerais; **Cerrado**: MS, Mato Grosso do Sul; MT, Mato Grosso; GO, Goiás, TO: Tocantins; **Pantanal**: MS*, Mato Grosso do Sul, MT*, Mato Grosso; **Amazônia**: TO, Tocantins; RO, Rondônia. **#,** Holotypes. **Note**: Some insects carried more than one *Angomonas* species/genotype
