# Supplementary material for: Genetic Diversity and Phylogenetic Relationships of Coevolving Symbiont-Harboring Insect Trypanosomatids, and Their Neotropical Dispersal by Invader African Blowflies (Calliphoridae)
Source: Front Microbiol. 2018 Feb 7;9:131. doi: 10.3389/fmicb.2018.00131 (PMC5808337; doi:10.3389/fmicb.2018.00131)
Supplement: Supplementary file 2 [file Table_2.PDF]

SUPPLEMENTARY TABLE 2

GenBank accession numbers of DNA sequences from *Angomonas* species and genotypes and their respective symbionts.

Species genotype	TCC code	GenBank accession number			
		V7V8 SSU rRNA	gGAPDH	ITS1 rDNA	GAPDH symbiont
A. desouzai					
	079E	HM593016	HM593020#	HM593027#	KY888335
	1279	HM593017	HM593021#	HM593029#	KY888336
	1310	KY888321	KY888307	-	-
	2214	MG682143	MG682170	-	MG682191
	ISC0012a	MG682144	MG682171	-	-
	2627	MG682145	-	-	MG682192
	2459	MG682146	-	-	MG682193
	ISC0075	-	-	-	MG682194
	ISC0051	-	-	-	MG682195
	ISC0010a	-	-	-	MG682196
	ISC0081	-	-	-	MG682197
	ISC0107	-	-	-	MG682198
A. ambiguus					
	1780	HM593015	HM593019#	HM593031#	KY888337
	1765	KY888322	KY888309	KY888316	KY888338
	ISC0004b	MG682147	MG682172	-	-
	ISC0011	MG682148	MG682173	-	-
A. deanei					
**Dea1**					
	036E	HM593011	HM593022	HM593034#	KY888339
	1743	HM593043	KY888312	HM593037#	KY888340
	2315	KY888323	-	-	KY888341
	263	AF038024#	EU079131#	-	-
	080E	HM593012#	EU079130#	-	-
	1752	MG682149	MG682174	-	-
	1757	MG682150	MG682175	-	MG682199
	1158	MG682151	-	-	MG682200
	ISC0010b	-	-	-	MG682201
**Dea2**					
	1759	KY888324	KY888310	KY888317	KY888342
	2332	KY888325	KY888311	KY888318	KY888343
	2455	KY888326	MG682177	-	KY888344
	2317	MG682152	MG682176	-	MG682202
**Dea3**					
	1445	HM593013	HM593023#	HM593036#	
	1639	KY888327	KY888313	-	KY888345
	1756	HM593014	HM593024#	HM593039#	KY888346
	ISC0011a	MG682153	MG682178	-	MG682212
	ISC0004a	MG682154	MG682179	-	
	1940	MG682155	MG682180	-	MG682203
	1884	MG682156	MG682181	-	MG682204
	1762	MG682157	MG682182	-	MG682205
	2018	MG682158	MG682183	-	MG682206
	2025	MG682159	MG682184	-	MG682207
	1715	MG682160	MG682185	-	-
	1923	MG682161	MG682186	-	-
	1763	MG682162	MG682187	-	-
	2046	MG682163	MG682188	-	-
	1760	MG682164	MG682189	-	-
	2454	MG682165	-	-	MG682208
	2534	MG682166	-	-	MG682209
	2705	MG682167	-	-	MG682210
	2706	MG682168	-	-	MG682211
	ISC0070	-	-	-	MG682213
	ISC0099	-	-	-	MG682214
	ISC0109	-	-	-	MG682215
	ISC0089	-	-	-	MG682216
	ISC0090	-	-	-	MG682217
	ISC0073	-	-	-	MG682218
	ISC0085	-	-	-	MG682219
**Dea4**					
	2446	KY888328	-	KY888319	KY888347
	2447	KY888329	KY888314	-	KY888348
	2448	MG682169	MG682190	-	-
	2450	KY888330	KY888315	KY888320	KY888349
	ISC0104	-	-	-	MG682220
